# Editorial for Special Issue “Phytochemicals in Cancer Chemoprevention and Treatment”

**DOI:** 10.3390/cimb47040289

**Published:** 2025-04-18

**Authors:** Wojciech Trybus, Ewa Trybus, Aneta Węgierek-Ciuk

**Affiliations:** Department of Medical Biology, Jan Kochanowski University of Kielce, Uniwersytecka 7, 25-406 Kielce, Poland; aneta.wegierek-ciuk@ujk.edu.pl

Despite significant progress in the treatment of cancer patients, modern oncological therapy faces numerous challenges that are primarily related to the lack of response to treatment caused by the resistance of cancer cells to chemotherapeutics. Another major problem is the emerging toxicity of therapy and its associated side effects. Hence, new, alternative treatments and compounds with anticancer potential are constantly sought. Phytochemicals found in plants, for example, and are currently being studied for their anticancer properties. The aim of this Special Issue was to present a range of compounds with anticancer potential in various experimental approaches to show their possible use in cancer treatments.

## 1. Introduction

According to statistics from the International Agency for Research on Cancer (IARC), nearly 20 million new cancer cases and 9.7 million cancer-related deaths were reported in 2022. It is estimated that about one in five people will develop cancer during their lifetime, while about one in nine men and one in 12 women will die from it. According to the forecast based on demographic data, the number of new cancer cases will reach 35 million by 2050 [1], which is a very serious social problem. The results of clinical and epidemiological studies clearly indicate that the principal factor contributing to the development and prevention of cancer is lifestyle. An incorrect diet, lack of physical activity, alcohol consumption, and smoking are the main factors inducing an increase in the incidence of various types of cancer [2].

The main methods to treat cancer are chemotherapy and radiotherapy supplemented by surgery, which, despite their constant use, also cause numerous side effects [3]. The main disadvantages of chemotherapy are possible cancer recurrences and toxic effects on non-targeted tissues, which in turn may limit the use of anticancer drugs and worsen the patient’s quality of life. Another significant cause of anticancer therapy failure is the innate and acquired resistance of cancer cells regulated, among others, by genetic and epigenetic factors that allow cancer cells to survive [4]. The basic feature of cancer cells is their ability to avoid the process of apoptosis, which results from the deregulation of molecular pathways contributing to the development of multidrug resistance. This applies to Bcl-2 family proteins, the p53 tumor suppressor, the overexpression of IAP controlling the activation of caspases, and the PI3K/AKT pathway, among others [5].

Therefore, new and alternative treatments and new groups of compounds with anticancer potential are sought, an example of which are numerous phytochemicals. The attempt to draw attention to the use of plant compounds in oncological therapy results from the fact that numerous studies have highlighted their anticancer properties [3], and herbal medicine is one of the oldest forms of therapy that has developed all over the world [6,7]. An example is Traditional Chinese Medicine, the oldest field in which recognizing the use of herbs for medicinal purposes is Materia Medica, which includes over 1800 entries with over 11,000 formulas providing full information about their medicinal use [7].

Over the years, many compounds have been isolated from plants that have become extremely important to medicine. Some key examples include taxoids and taxol, discovered in the 1960s and 1970s, isolated from the bark of *Taxus brevifolia*, which remains one of the most effective methods of treating breast and ovarian cancer [8]. Another example is the alkaloid camptothecin, isolated from the bark of *Camptotheca acuminata*, whose anticancer effect is based on the inhibition of topoisomerase I [9]. Another breakthrough was the discovery of the *Vinca* alkaloids in the 1960s, i.e., vinblastine and vincristine, which have made an equally substantial contribution to oncological therapy. Vincristine is a chemotherapeutic drug used to treat acute lymphoblastic leukemia, Hodgkin’s and non-Hodgkin’s lymphomas, Wilms’ tumor, rhabdomyosarcomas, retinoblastoma, neuroblastoma, and various brain tumors. Its analog, vinblastine, is used in chemotherapy for Hodgkin’s lymphoma, Langerhans cell histiocytosis, and brain gliomas [10].

According to Choudhari et al. (2020), clinical trials on the use of phytochemicals in anticancer therapy are currently focusing on three aspects: improving the response of cancer cells to standard chemo- and radiotherapy, reducing the serious side effects of standard therapy, and looking for undesirable interactions with standard therapy [11].

## 2. Selected Phytochemicals with Anticancer Properties

In this summary, we focus on several groups of phytochemicals to highlight their potential anticancer properties. An example of compounds whose anticancer properties have been demonstrated in numerous literature reports are alkaloids, which are the main bioactive chemical components of the Berberis species responsible for various pharmacological effects of both the whole extract and isolated single compounds [12]. The most well-known alkaloid with anticancer effects is berberine, the mechanism of which is based on scavenging free radicals, inducing apoptosis, blocking the cell cycle, or inhibiting angiogenesis, among others [13].

In the study by Miłek et al. (2024), it was demonstrated that extracts from ornamental barberry twigs, compared to pure berberine sulfate administered in an analogous dose, present greater cytotoxicity towards HaCaT, A375, and Caco-2 cell lines. At the same time, the team showed that ornamental barberry twigs, easier to obtain with the controlled cultivation of this plant than common barberry roots, can be used as a raw material for the isolation of berberine for the pharmaceutical industry [14].

Magnolia-derived lignans, such as honokiol and magnolol, may prove to be effective compounds in treating patients with head and neck squamous cell carcinoma. A study by Klesz et al. (2024) confirmed that these compounds can limit the viability of cancer cells by regulating the cell cycle and apoptosis, which are probably related to changes in the expression level of BIRC5 and CDKN1A. It is also interesting that the tested compounds showed cytotoxicity in FaDu cisplatin persister cells, which is key to overcoming chemoresistance [15].

Statistics show that cervical cancer accounts for 70% of all cancer cases. In 2018, more than 560,000 new cases of cervical cancer were diagnosed worldwide, with more than 300,000 resulting in death. Of these, more than 80% occurred in low- and middle-income countries, such as South Africa, India, China, and Brazil, with the main contributing factor to the development of cervical cancer being infection with the sexually transmitted human papillomavirus [16].

Hence, it is important to pay attention to new methods of treatment, including the use of compounds with significant anticancer activity in relation to this type of cancer. An example is cacalol acetate, which is a derivative of cacalol, a sesquiterpene isolated from *Psacalium decompositum*. Rostro-Alonso and colleagues (2024) were the first to prove that this derivative has cytotoxic, antiproliferative, proapoptotic, and antimigratory effects in relation to the HeLa cell line studied [17].

Alaouna et al. (2024) demonstrated the efficacy of the South African plant *Tulbaghia violacea* in the treatment of metastatic triple-negative breast cancer (TNBC) [18]. This is an interesting report since TNBC is most often an invasive high-grade ductal carcinoma, which is characterized by an aggressive clinical phenotype and lack of estrogen and progesterone receptor expression and human epidermal growth factor receptor 2 (HER2) expression. It is also one of the most common subtypes of breast cancer in women with mutations in the BRCA1 gene [19,20]. It was shown that an aqueous extract containing anticancer compounds (DDMP, 1,2,4-triazine-3,5(2H,4H)-dione, vanillin, schizandrin, taurolidine and α-pinene) affected the adhesion, invasion, and migration of MCF-10A and MDA-MB-231 cell lines. Alterations in genes related to angiogenesis, metastasis, and proliferation were also demonstrated, with reduced activity in growth receptor signaling, angiogenesis, and cancer-related pathways (Wnt, Notch and PI3K pathways), which is highly relevant in the development of therapeutics targeting TNBC metastases [18].

Ashwagandha (*Withania somnifera* L. Dunal), in which withaferin A and withanolides are considered promising anticancer compounds, also shows great promise in the treatment of breast cancer, especially with estrogen receptor/progesterone receptor (ER/PR)-positive and triple-negative breast cancer [21]. Numerous studies also indicate its anti-inflammatory, antimicrobial, cardioprotective, and antidiabetic properties [22].

Anticancer therapy can also be supplemented by alternative medicinal compounds obtained from marine flora, an example of which are micro- and macroalgae containing various types of bioactive molecules, including carotenoids and various forms of polysaccharides with anticancer activity, which act by inducing apoptosis and inhibiting the cell cycle and proliferation [23]. The interest in microalgae stems from the fact that they are a component of the daily diet and a food additive, mainly in East Asian countries. Active metabolites of algae known for their anticancer and biocidal effects are also of great interest [24]. Algae are also a source of antioxidant compounds, including carotenoids, vitamins, and phenolics, used not only in medicine but also in other branches of the pharmaceutical industry [25].

Compounds with high anticancer potential also include polysaccharides, which differ from each other in both structure and toxicity profile [26]. An example is the extracellular polysaccharides (EPS) studied by Toshkova-Yotova et al. (2024) isolated from a new Bulgarian strain of the green microalga *Coelastrella* sp. BGV, which show anticancer activity in cervical and breast cancer cells based on the induction of apoptosis, inhibition of the cell cycle, and antiproliferative activity demonstrated in a wound healing test. Interestingly, the authors did not demonstrate any cytotoxic effect on normal cells (BALB/3T3 and HaCaT lines); hence, they conclude that this new microalgae strain, as a source of EPS with selective anticancer activity, should be studied in terms of its pharmacological and biotechnological potential [27].

Numerous studies have shown that polyphenols are also phytochemicals found in many plant-based diets with chemopreventive effects on various cancers. Interest in these compounds is constantly growing because studies indicate a link between a diet rich in soy and cancer avoidance, which has been linked to the presence of genistein—a phenolic component of soy [28]. Preclinical studies have shown multidirectional effects of genistein based on antioxidant, anti-inflammatory, antibacterial, and antiviral properties, and its mechanism of anticancer action has been described on many cancer cell lines [29]. However, Banyś et al. (2024) have shown that supplementation of animals with genistein in macro, micro, and nano forms increases the intensity of the neoplastic process, the level of metalloproteinase-9, and the expression of the MMP-9 gene, and significantly reduces the level of eicosanoids (HETEs, HODE, and HEPE) [30]. Also, a study by Ju et al. suggests that consuming genistein-containing products may be dangerous for postmenopausal women with estrogen-dependent breast cancer. Genistein has been shown to act in an additive manner at low levels of 17β-estradiol, stimulating estrogen-dependent tumor growth in vivo [31].

The use of an appropriate diet may also affect the activity of key multidrug resistance transporters (MRP2, BCRP, and P-gp) influencing the effects of chemotherapy. According to Brodzicka et al. (2024), such phytochemicals include catechins, flavonoids, resveratrol, curcumin, terpenoids, sterols, and alkaloids, which, by modulating the activity of MDR transporters, impact the effectiveness of chemotherapy. The authors conclude that, in the context of improving treatment results and reducing side effects, attention should be paid to the interaction between diet and the drugs used in cancer therapy [32].

Another group of biologically active compounds are ribosomally synthesized and post-translationally modified peptides (RiPP) from plants. In their review, Hwang et al. (2024) paid special attention to rubipodanin A and mallotumide A–C, which exhibit low nanomolar IC50 values against many types of cancer cells and emphasize the importance of plant RiPP in developing innovative methods of cancer treatment [33].

Rosmarinic acid, apigenin, and thymoquinone also have chemopreventive potential, inhibiting the growth of cancer cells and modulating key signaling pathways involved in cancer development [34].

Therefore, there is a constant need to study new groups of plant compounds for their anticancer properties and analyze and discover their mechanisms of action in both in vitro and in vivo systems to develop new drugs with anticancer properties.

When discovering drugs based on plant products, attention should be paid to many important factors, including the availability and effectiveness of the plant material or the compound being tested, the ability to modify the structure, the molecule size, the stability of the compounds, and their toxicity [35]. Hence, molecular-level studies are important to determining the chemical components of plants, their chemical configuration, the process of biosynthesis and degradation, natural distribution, or bioactivity [36]. However, the main problem in the research on phytochemicals at the molecular level is the lack of a complete understanding of their interactions with various signaling molecules, which can be overcome by using techniques such as molecular docking, QSAR modeling (Quantitative structure–activity relationship model), or cross-linked pharmacology. Additionally, the use of LC-MS and LC-NMR techniques can significantly contribute to accelerating the identification of compounds [11], and thus the possibility of using phytochemicals as new drugs.

## 3. Conclusions

In this Special Issue of the journal *Current Issues in Molecular Biology*, the latest research on various phytochemicals with potential anticancer properties is collected and their mechanism of action is presented both in vitro and in vivo. We hope that the presented results and data included in the review articles will make a significant contribution to the development of modern phytotherapy and will encourage further research on the presented phytochemicals for their future use in oncology.
